# Genotype characterization of human papillomavirus in women infected and uninfected with HIV in Ouagadougou, Burkina Faso

**DOI:** 10.1186/1742-4690-9-S1-P36

**Published:** 2012-05-25

**Authors:** Florencia W Dkigma, Djeneba Ouermi, Tani Sagna, Charlemagne Ouedraogo, Cyrille Bisseye, Moctar Zeba, Simplice D Karou, Virginio Pietra, Jean-Baptiste Nikiema, Jacques Simpore

**Affiliations:** 1 Centre de Recherche Biomoléculaire « Pietro Annigoni » CERBA/LABIOGENE, Ouagadougou, Burkina Faso

## Introduction

Our study aims to compare the prevalence and genotypes of HPV in HIV-positive and negative women in Ouagadougou, where HIV and HPV prevalence are respectively estimated at 4.0% and 24%.

## Materials and methods

The study involved 410 women: 205 HIV-positive followed by two sites of HIV/AIDS care in the eastern outskirts of Ouagadougou, matched by age with 205 HIV-negative consulting the gynaecological services in the same area. HPV genotyping was done by PCR followed by reverse hybridization on nitrocellulose strips with the kit "STAR HPV Blot” (Diatech ®, Italy).

## Results

Prevalence of HPV was: 25.4% among HIV-negative women and 59.0% among HIV-positive women (p <0.01). Means of age was respectively 33.6 (SD±8.6) versus 33.4 (SD±6.4). Prevalence of HPV subtypes at low risk of oncogenicity (6,11,LR) among HIV-positive women was 9.8% and 9.3% in the control group (p=ns). Prevalence of HPV subtypes at high risk of oncogenicity was significantly(p<0.01) higher among HIV-positive women for subtypes 50’S (22.0% versus 9.3%, OR=2.8 ; CL95% 1.5-5.1), 18 (20.0% versus 2.9%, OR=8.3 ; CL95% 3.2–23.0), 30’S (17.6% versus 1.5%, OR=14.3 ; CL95% 4.1–60.1) and HR ( 7.3%, versus 1.0%, OR=8.0 ; CL95% 1.7–52.1) while no significant difference was observed for high risk subtypes 16 and 45. Co-infections by two or more subtypes at high risk were detected in 27/205 (13.2%) HIV-positive women and 3/205 (1.5%) HIV-negative women (p<0.01; OR 10.2; CL95% 2.9-43.5). Among HIV-positive women, prevalence of oncogenic subtypes was significantlycorrelated with a lower CD4 count (P = 0.05).

## Conclusions

HIV-positive women are at high risk of coinfection by HPV oncogenic subtypes. This study confirms the need to integrate the screening of cervical cancer in HIV care protocols in Burkina Faso. Further investigations should be continued for the establishment of vaccine that matches all genotypes circulating in the country.

